# Feeling matters: perceived social support moderates the relationship between personal relative deprivation and depressive symptoms

**DOI:** 10.1186/s12888-021-03334-8

**Published:** 2021-07-12

**Authors:** Sibo Zhao, Li Peng

**Affiliations:** School of Sociology and Psychology, Central University of Finance and Economics, 39 South College Road, Beijing, 100081 China

**Keywords:** China, Personal relative deprivation, Depressive symptoms, Perceived social support

## Abstract

**Background:**

Little research describes the mechanisms underlying depressive symptoms and personal relative deprivation in Chinese populations.

**Methods:**

In this study, the respondents were (*N* = 914) residents of Beijing (17–59 years old) and robust multiple linear regressions were used to assess the main relationship between relative deprivation and depressive symptoms and social support as a potential moderator for that relationship.

**Results:**

Individuals who reported higher personal relative deprivation had greater depressive symptoms than those who reported lower personal relative deprivation. Perceived social support buffered the relationship between depressive symptoms and personal relative deprivation.

**Conclusions:**

The findings of this current study demonstrate the importance of relative deprivation for psychological strain and income in explaining how socioeconomic indices correlate with depressive symptoms. They also demonstrate the need to acknowledge the interaction of perceived social support and personal relative deprivation for influencing depression.

**Supplementary Information:**

The online version contains supplementary material available at 10.1186/s12888-021-03334-8.

## Background

Depressive symptoms are usually regarded as negative emotional experiences such as those which might ensue after a series of negative life events. Mounting evidence indicates that depressive symptoms are associated with increased and prolonged psychological crises and health-risky behavior, and one of their most extreme consequences is suicide [1, 2]. The global prevalence of depression (lifetime prevalence of depression without comorbid anxiety disorder) is about 5.3% [3], and the prevalence of depression in mainland China is about 6.8% [4].

### The association between depressive symptoms and relative deprivation

A number of researchers have taken depressive symptoms as an outcome of health inequality [5, 6], and relative deprivation has been used as an indicator of such inequality or a predictor of mental health in a variety of studies [7, 8]. The theoretical framework of relative deprivation, which was originally elaborated by WG Runciman [9], indicated that relative deprivation derives from a comparison of one’s own economic status and that of others to find that the comparer is worse off. The *Relative Deprivation Hypothesis* suggests that inequality motivates individual-level socioeconomic comparisons, and these lead to worsened social relations, greater stress, and resulting poorer mental health and well-being, including depressive symptoms (reviewed in [10–12]). Nevertheless, though substantial evidence has supported the relative deprivation hypothesis, it remains controversial concerning the appropriate measurements of individual relative deprivation and the understanding of the subsequent contradictory results.

### The measurements of relative deprivation

One controversy is about the measurements of relative deprivation. Many previous studies have used one economic measure instead of comprehensive measure to assess relative deprivation. Specifically, some have focused on the magnitude of income difference, while others were concerned about income rank. For example, researchers have taken a measure of relative income with reference to a mean income level of an individual, state, or country [13, 14], or developed a variety of indexes (e.g., the Yitzhaki Index, the Podder Index, the Carstairs Index, and the Townsend Index), which usually incorporated a few broader social factors, such as unemployment rate, to quantify the income inequality in comparison to the broader population (reviewed in [8, 15, 16]). However, the findings on mental health and relative deprivation of income yield inconsistent results due to the use of different measures and the selection of different reference groups [17]. Consequently, a new line of subsequent psychosocial researches has focused on the relationship between mental health and income ranking, indicating that the income rank alone constitutes a better proxy variable for relative deprivation on psychosociology [18–20]. Yet, despite the concept of relative deprivation from an economic perspective is well known, personal relative deprivation is also considered to be a psychological consequence of inequality. This consequence is based not only on single item comparisons like income or social status but multidimensional comparisons as well (including personal competence, income, and place on the social ladder). Thus, the specific operationalized measurement of relative deprivation would differ across cultural context [21]. For example, since the 1980s, economic reforms in China have caused important changes in urban and rural families, which are closely related to personal relative deprivation in different ways [22]. Therefore, in this study, we will not only measure the relative deprivation concerning individual characteristics like income, competence and social status, but also use a comprehensive measurement, taking family background into account.

### The objective and subjective relative deprivation

Another controversy is that whether objective status of relative deprivation can reflect the subjective feeling of being deprived. Subjective feeing is about an individual’s own sense of life-time achievement and socioeconomic status, which may be misaligned with objective indicators. For example, MK Whyte [23]) pointed out that compared to people in other countries, people in China responded to the inequality with a more positive attitude, and the negative feeling about inequality was less than expectation in spite of the increasing aggravation of social inequality after the economic reforms. On this basis, some researchers paid more attention to the individual-level subjective perception of inequality, and examined the association between that and poorer mental and physical health [24, 25]. For example, Demakakos and colleagues [26] found that subjective social status significantly predicted a number of health-related outcomes in old age, including depression. Zhang and colleagues, using their Psychological Strain Scale (PSS), posited four types of strains that predict mental health outcomes (including anxiety, depression, and suicidal ideation), one of which is relative deprivation strain, measured with a 10-item scale that assesses perceived differences between the respondent’s own status and the perceived status of others [27]. Hence, compared to the previous research that focuses on objective relative deprivation, this study pays far more attention on exploring the effect of subjective relative deprivation on mental health.

Only a handful of empirical papers have examined subjective feelings of relative deprivation and depressive symptoms [24, 25], and there are still few studies that have used both single and comprehensive measures to assess personal relative depravation. Relative income deprivation measures a single aspect of social inequality, while a multidimensional comparison of relative deprivation assesses a range of aspects of a person’s life and is more likely to vary in different personal scenarios. It is therefore possible that the association between a single measure of personal relative deprivation and depressive symptoms is different from the association between comprehensive measures and depressive symptoms, especially after absolute personal income is controlled for. Therefore, the first attempt of this study is to compare the effect of single and comprehensive measure of personal relative deprivation on depressive symptoms.

### Potential moderating effect of perceived social support

Perceived social support is an influential force buffering the relationship between psychosocial stress and mental health, that individual perceived via one’s social network to protect one from negative events or improve one’s well-being [28]. Unfortunately, few recent studies have examined how perceived social support contributes to mechanisms underlying the relationship between personal relative deprivation and depressive symptoms. Previous studies were found that perceived social support is negatively related to depressive symptoms [29, 30]. College students with lower levels of social support have been found to be more likely to exhibit high levels of depression, which may also increase the risk for suicidal behavior [31, 32]. Social support is also considered as moderating variable that explains the relationship between the two variables. Zhang and Lin [33] found that social support serves a moderating function in suicides of rural youth in China, such that high social support had a protective effect for individuals with low impulsivity. In this circumstances, social support might help to buffer the shock of personal relative deprivation to mental health, and specifically, help to buffer depressive symptoms related to personal relative deprivation.

The limited evidence suggests that personal relative deprivation is associated with poorer mental health. We investigated the relationships between personal relative deprivation, social support, and depressive symptoms in a more comprehensive manner. In this study, we propose a conceptual model to describe the moderation exerted by the intermediary variable of social support on the relationship between personal relative deprivation and depressive symptoms. This study examines the following research hypotheses, grounded in the literature: (1) increased personal relative deprivation level is positively associated with increased depressive symptoms, and social support level is negatively associated with depressive symptoms; (2) perceived social support moderates the relationship between depressive symptoms and relative deprivation in income and relative deprivation in psychological strain.

## Methods

### Sampling and procedure

The data for the current study were obtained from questionnaires distributed among residents of Beijing in 2017. The survey was conducted by Questionnaire Star (https://www.wjx.cn/app/survey.aspx), a professional online survey company. The survey is available as supplementary material. The Principal Investigator signed a contract with the Questionnaire Star to specify the cost of the survey and how the responses would be kept confidential. On average, more than 1 million people fill out questionnaires on the platform of Questionnaire Star every day, and Questionnaire Star will randomly invite some of them to join the sample base. Professional investigator from Questionnaire Star was recruited to execute the investigation program, including sampling and marketing, etc. The respondents were limited to the current residents of Beijing aged from 17 to 59, and Questionnaire Star will provide compensation after the questionnaire is completed. The quality control methods undertaken were as follows:

1. the questionnaire was only distributed to users who meet selection requirements (aged 17–59 and resided in Beijing);
2. screening items were set to verify that participants met the sample qualifications (e.g., if a respondent did not meet the selection requirements, then the investigation would be terminated);
3. online systems were used to monitor the process; these took account of the IP addresses, tracked which electronic devices were used, and administered trap items, time limits, and sampling procedures;
4. after the completion of all questionnaires, a quality check was performed to assess the completeness, formatting, and effectiveness of each of the data records.

A total of 950 responses were investigated, and 12 were eliminated due to incompletion, giving a response rate of 98.7%. The sample for the current study was 914 because some respondents had missing values in the dependent or independent variables.

### Ethical approval

The research protocol of the study was formally approved by the Institutional Review Board of the Ethics Committee at the University where the Principal Investigator is affiliated. All participants provided written informed consent prior to completing the study self-report instruments and did not provide identifying information on any of the questionnaires.

### Measurements

#### Outcome (depression)

The Center for Epidemiologic Studies Depression Scale (CES-D) was used to evaluate depressive symptoms [34]. This self-report scale includes 20 items, each assessed on an 8-point Likert-type scale, with responses ranging from 0 to 7, representing how many days the participants have experienced depressive symptoms over the past week. Consistently with previous studies, this 8-point scale was re-coded into a 4-point scale, as follows: 0 = 0 days, 1 = 1–2 days, 2 = 3–4 days, and 3 = 5–7 days. The total points for all items (ranging from 0 to 60) was computed to indicate the level of depression. The four inverted items in the scale (items 4, 8, 15, and 20) were reversed before the tally. The good internal consistency of the scale for this sample was confirmed with Cronbach’s alpha (α = 0.9311).

#### Indicator (relative deprivation)

##### Relative deprivation in psychological strain

A slightly modified version of the relative deprivation subscale of the PSS was used to evaluate the level of relative deprivation [27]. For this, the respondents were asked to assess ten statements regarding situations they experienced in their lives, such as “Compared to other families in my community, my family is poor,” and “I believe I am good enough, but am dissatisfied with treatment from others.” Subjects rate their responses on the following five-point scale: 1 = never, 2 = rarely, 3 = maybe, 4 = often, and 5 = yes. The total score (ranging from 10 to 50) indicates the level of psychological strain due to relative deprivation, with higher scores associated with higher relative deprivation. The Cronbach’s alpha coefficient for the PSS subscale was α = 0.923.

##### Relative income deprivation

We designed a four-item instrument to assess respondents’ perceptions of relative lack of income compared to four groups as their references: friends, colleagues, occupation peers, and significant others. Each item was rated on a 5-point Likert-type scale: 1 = not at all satisfied, 2 = not very satisfied, 3 = generally satisfied, 4 = somewhat satisfied, and 5 = completely satisfied. The points were reverse coded, and the total score (ranging from 4 to 20) was used for analysis. Higher the scores indicated higher relative deprivation. The Cronbach’s alpha coefficient for this scale was α = 0.806.

#### Potential moderator (perceived social support)

The Multidimensional Scale of Perceived Social Support (MSPSS) was used to measure social support [35]. The Chinese 12-item version of the scale has been tested and found to have sound validity and reliability among Chinese adolescents [28]. The answers ranged from 1 = strongly disagree to 7 = strongly agree. The total score (ranging from 12 to 84) was analyzed in the present study. High internal consistency was found (Cronbach’s α = 0.9425).

#### Control variables

Sex was assessed as a binary variable, with 1 = male and 0 = female. Age was computed by subtracting the date of birth from July 2017. Marital status was coded as 0 = others (including cohabitation, separated but not divorced, divorced, and widowed), 1 = single, or 2 = married (including married and remarried). Education was coded as 0 = college degree and below (including no formal education, elementary school, middle school, vocational high school, high school, technical secondary school, technical school, and college) or 1 = bachelor’s degree and above (including university completion, graduate degree, and above). Occupation type (unclassifiable types were excluded, e.g., student and housewife) was converted to match the International Socioeconomic Index (ISEI), a general measure that evaluates the conversion capacity of occupations in terms of the substitutability of human resources and the potential payoff, where higher ISEI scores indicate higher socioeconomic status [36]. Monthly personal income was transformed to a logarithmic scale and controlled for in the model, which was used to measure objective relative deprivation. Location of origin was a binary variable, with 1 indicating those from urban China and 0 indicating those from rural China.

### Analysis

All data were analyzed with STATA 16.0. For all continuous variables, t-tests were performed, and chi-square tests were performed for non-continuous variables. Pearson’s correlation was computed to examine the relationship between variables.

Robust multiple linear regressions were performed using the iteratively reweighted least square (IRLS) method to examine the associations between the dependent variable and the explanatory variables. IRLS does not impose an assumption of a normal distribution in a sample but instead assigns an analysis weight that is yielded from an iterative algorithm for each observation to deal with non-normally-distributed sample, meaning that the estimation is more effective and robust than the ordinary least square (OLS) model.

## Results

### Description of demographic characteristics

The sample contains 914 observations of Beijing residents who completed the entire questionnaire. Table 1 illustrates the respondents’ socio-demographic characteristics by the likely presence of clinically significant depression (with the cutoff score of 16). Ages ranged from 17 to 59 years, and 51.81% of respondents were male.

**Table 1:** Demographic descriptions for study variables and their depression comparison

Variable	Total (***n*** = 914) M ± SD/f (%)	No Depression (***n*** = 390) M ± SD/f (%)	Depression (***n*** = 524) M ± SD/f (%)	t/χ^**2**^
RD in psychological strain	25.86 ± 8.01	21.95 ± 7.01	28.78 ± 7.46	−14.017^***^
Social support	62.05 ± 9.90	66.98 ± 7.21	58.37 ± 10.04	14.386^***^
RD in income	18.23 ± 3.28	17.43 ± 3.04	18.83 ± 3.34	−6.508^***^
Ln (monthly-income)	9.06 ± 0.50	9.16 ± 0.49	8.99 ± 0.49	5.084^***^
ISEI	54.06 ± 14.28	54.27 ± 13.44	53.91 ± 14.88	0.384
Sex				0.346
Female	442(48.36)	193(43.67)	249(56.33)	
Male	472(51.64)	197(41.74)	275(58.26)	
Age				2.012
17–29	333(36.43)	132(39.64)	201(60.36)	
30–39	414(45.30)	185(44.69)	229(55.31)	
40–59	167(18.27)	73(43.71)	94(56.29)	
Marital				4.311
Single	216(23.63)	91(42.13)	125(57.87)	
Married	625(68.38)	276(44.16)	349(55.84)	
Others	73(7.99)	23(31.51)	50(68.49)	
Education				4.815^*^
College and below	486(53.17)	191(39.30)	295(60.70)	
Bachelor and above	428(46.83)	199(46.50)	229(53.50)	
Residence				24.830^***^
Rural	146(15.97)	35(23.97)	111(76.03)	
Urban	768(84.03)	355(46.22)	413(53.78)	

*Note*. *p < 0.05; **p < 0.01; ***p < 0.001

The t-test or the chi-square test was used to test the significance of the difference in diagnosis of depression in relative deprivation scores in psychological strain and income, MSPSS scores (social support), and other variables for the study. The depressive respondents reported higher relative deprivation scores in psychological strain and income, and they also had lower perceived social support scores and monthly income than no depression respondents. Among the 914 respondents, 68.38% were married and 23.63% were single. There were no significant differences in marital status between depressive and no depression respondents, *p* > 0.05. More than half of respondents had an educational level of college or below (53.17% vs. 46.83%), and respondents with a lower diploma tended to be depressive (*p* = 0.028). More respondents were from urban than rural areas (84.03% vs. 15.97%), which is consistent with the distribution of population in Beijing overall, and more respondents from rural areas suffered from depressive symptoms (*p* < 0.001).

### Correlation analysis

We performed correlation analysis between depressive symptoms and other major variables. Pearson’s correlation coefficients and the Sidak-adjusted significant level of counterparts are shown in Table 2. In the correlation matrix it can be seen that depressive symptoms were significantly positively associated with relative deprivation in psychological strain (*r* = 0.534, *p* < 0.001) and significantly negatively associated with social support (*r* = − 0.474, *p* < 0.001). Additionally, depressive symptoms had a weakly positive correlation with relative deprivation in income (*r* = 0.224, *p* < 0.001). Furthermore, respondents with greater social support scores tended to have lower scores for relative deprivation in psychological strain (*r* = − 0.317, *p* < 0.001) and lower CES-D scores (*r* = − 0.474, *p* < 0.001), indicating a potential interaction mechanism between social support and relative deprivation in psychological strain.

**Table 2:** Correlations among major study variables

Variables	Depression	RD in psychological strain	Social support	RD in Income	Ln (monthly-income)
Depression	1				
RD in psychological strain	0.534^***^	1			
Social support	−0.474^***^	−0.317^***^	1		
RD in Income	0.224^***^	0.253^***^	− 0.457^***^	1	
Ln (monthly-income)	−0.199^***^	− 0.312^***^	0.240^***^	− 0.112^**^	1

*Note*. *p < 0.05; **p < 0.01; ***p < 0.001

### Robust regression model

Table 3 presents the results of the multiple regression models. In this part, we conducted five models to test the assumptions of this study. Model 1(*R*^2^ = 0.112, F (9,904) =12.72, *p* < 0.001) was conducted to evaluate the effect of objective relative deprivation on depressive symptoms. We used Model 2 (*R*^2^ = 0 .162, F (10,903) =17.53, *p* < 0.001) and Model 3 (*R*^2^ = 0.329, F (10,903) =44.45, *p* < 0.001) to evaluate the effect of subjective relative deprivation on depressive symptoms and make a comparison between the effect of relative deprivation in income and relative deprivation in psychological strain on depressive symptoms. In Model 4 (*R*^2^ = 0.323, F (12,901) =35.84, *p* < 0.001) and Model 5 (*R*^2^ = 0.435, F (13,900) =53.39, *p* < 0.001), we evaluated the moderating effect of perceived social support.

**Table 3:** Robust multiple regression for depressive symptoms

Variable	Depression									
	Model1		Model2		Model3		Model4		Model5	
	β (S.E.)	***P***	β (S.E.)	***P***	β (S.E.)	***P***	β (S.E.)	***P***	β (S.E.)	***P***
RD in psychological strain					0.690 (0.039)	< 0.001			0.569 (0.038)	< 0.001
Perceived social support							−0.500 (0.035)	< 0.001	−0.400 (0.033)	< 0.001
RD in income			0.771 (0.101)	< 0.001			0.189 (0.100)	0.061	−0.042 (0.094)	0.655
Ln (monthly-income)	−4.226 (0.753)	< 0.001	−3.729 (0.728)	< 0.001	−0.791 (0.679)	0.244	−2.088 (0.653)	0.001	−0.133 (0.623)	0.831
ISEI	0.054 (0.025)	0.034	0.044 (0.024)	0.069	0.056 (0.022)	0.011	0.025 (0.021)	0.239	0.045 (0.020)	0.025
Sex (Female)										
Male	1.150 (0.726)	0.113	0.756 (0.699)	0.280	−0.377 (0.632)	0.551	0.564 (0.621)	0.364	−0.343 (0.577)	0.552
Age (40–59)										
17–29	1.086 (1.118)	0.332	1.920 (1.084)	0.077	2.091 (0.970)	0.031	2.769 (0.966)	0.004	3.585 (0.895)	< 0.001
30–39	−0.175 (0.981)	0.858	0.368 (0.949)	0.698	0.457 (0.852)	0.591	0.619 (0.842)	0.462	1.331 (0.782)	0.089
Marital (Others)										
Single	−5.812 (1.421)	< 0.001	−5.361 (1.366)	< 0.001	−5.766 (1.231)	< 0.001	−3.159 (1.212)	0.009	−4.441 (1.122)	< 0.001
Married	−4.963 (1.317)	< 0.001	− 4.379 (1.267)	0.001	− 4.361 (1.141)	< 0.001	−0.870 (1.136)	0.444	−1.968 (1.049)	0.061
Education (College and below)										
Bachelor and above	−0.902 (0.726)	0.214	−0.920 (0.698)	0.188	−0.475 (0.629)	0.450	0.052 (0.620)	0.933	0.015 (0.574)	0.979
Residence (Rural)										
Urban	−5.949 (0.970)	< 0.001	− 5.912 (0.933)	< 0.001	−4.098 (0.844)	< 0.001	− 4.448 (0.831)	< 0.001	−3.352 (0.770)	< 0.001
RD in income # perceived social support							−0.020 (0.008)	0.012		
RD in Psychological strain # perceived social support									−0.008 (0.003)	0.016
Constant	8.349 (1.854)	< 0.001	7.421 (1.784)	< 0.001	6.675 (1.609)	< 0.001	2.245 (1.607)	0.163	2.784 (1.483)	0.061
*R*^2^	0.112 (F = 12.72, *P* < 0.001)		0.162 (F = 17.53, *P* < 0.001)		0.329 (F = 44.45, *P* < 0.001)		0.323 (F = 35.84, *P* < 0.001)		0.435 (F = 53.39, *P* < 0.001)	

In Model 1, controlling for other variables, increased absolute income indicating lower level of objective relative deprivation, was significantly negatively associated with depressive symptoms (*p* < 0.001). In addition, respondents from urban areas reported lower depressive symptoms than respondents from rural areas (*p* < 0.001), and respondents who were single or married reported lower depressive symptoms than people with other marital status (*p* < 0.001). The significantly positive coefficient of ISEI indicated that higher ISEI was associated with stronger depressive symptoms. One possible reason might be that those who had a job with higher ISEI were undertaken more occupational stress, which was positively associated with higher depressive symptoms as shown in the previous study [37]. However, no evidence was found to support significance of effects of age, sex, or education level on depressive symptoms in Model 1.

Subsequently, we separately added relative deprivation in income (Model 2) and relative deprivation in psychological strain (Model 3) into Model 1 to compared if the relationship with depressive symptoms differs between these two measures of relative deprivation. It was found that, after controlling for other variables, subjective relative deprivation both in income and psychological strain had a significantly positive association with depressive symptoms (*p* < 0.001). In Model 2, higher levels of depressive symptoms were associated with higher income relative deprivation and lower income (*p* < 0.001). The coefficient of determination in Model 3 was much higher than that in Model 1 and Model 2. However, the coefficient of the absolute income was no longer statistically significant, indicating that the subjective relative deprivation in psychological strain had a suppression effect on the association between objective relative deprivation and depressive symptoms. In addition, higher depressive symptoms were observed among respondents aged 17–29 years (*p* = 0.031) than in the reference group (aged 40–59 years), whereas the difference in depressive symptoms between those aged 30–39 years and the reference group were nonsignificant (*p* = 0.591).

Finally, we assessed the moderation effect using Model 4 and Model 5. In Model 4, we included perceived social support and its interaction items with income relative deprivation based on Model 2 to assess the hypothesis of moderation. The model’s coefficient of determination increased significantly from 0.162 to 0.323. After other variables were controlled, it was found that the interaction item of perceived social support and income relative deprivation was negatively related to depressive symptoms (*p* = 0.012). In Model 5, we assessed the interactions between relative deprivation in psychological strain and perceived social support based on Model 3 to test whether the effect of relative deprivation in psychological strain on depressive symptoms was moderated by perceived social support. The model’s coefficient of determination increased significantly from 0.329 to 0.435. The result for the interaction term was statistically significant, indicating that perceived social support did reduce the marginal predictive effect of relative deprivation in psychological strain on depressive symptoms (*p* = 0.016). However, the association between relative deprivation in income and depressive symptoms was not statistically significant after controlling for other variables.

## Discussion

We explored the association between personal relative deprivation, perceived social support, and depressive symptoms in China. We were particularly interested in testing whether perceived social support buffers the relationship between depressive symptoms and personal relative deprivation, with both single and comprehensive measures of personal relative deprivation. We found that individuals who reported higher personal relative deprivation had higher depressive symptoms than those who reported lower personal relative deprivation. Further, perceived social support had a negative association with depressive symptoms, providing supporting evidence for the first hypothesis. These results were consistent with those of previous research.

To explore heterogeneity among individuals further, we added two types of personal relative deprivations and two interactions between perceived social support and relative deprivations to our models. These models provide qualified support for our second hypothesis. Model 4 showed that the positive direct effect of relative deprivation in income was buffered by perceived social support. Model 5 indicated that the positive direct effect of relative deprivation in psychological strain was statistically significant even with a significant interaction term in the model, indicating that individuals with a relative deprivation in psychological strain and perceived lower social support had a higher risk of depressive symptoms than those who perceived higher social support.

These results constitute new evidence for the association between personal relative deprivation and depressive symptoms. First, our findings support a significant positive association between personal relative deprivation and depressive symptoms and highlighted the use of comprehensive measures of subjective relative deprivation. Assuming that relative deprivation in income is a better predictor, we would expect an increase in relative deprivation in income to be associated with an increase in depressive symptoms even after controlling for other variables.

**Fig. 1:**
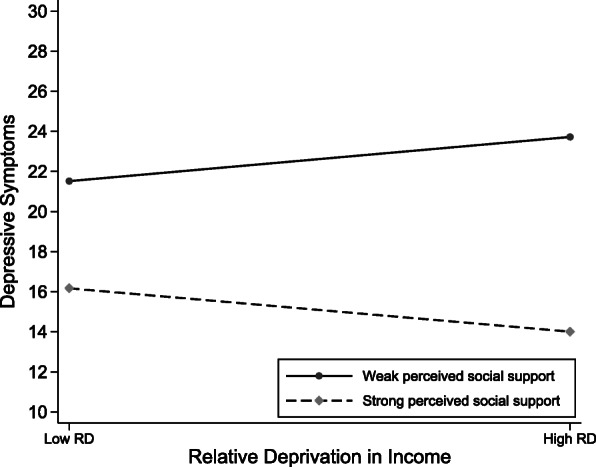
Graph of the moderating effects of perceived social support on relative deprivation in income

**Fig. 2:**
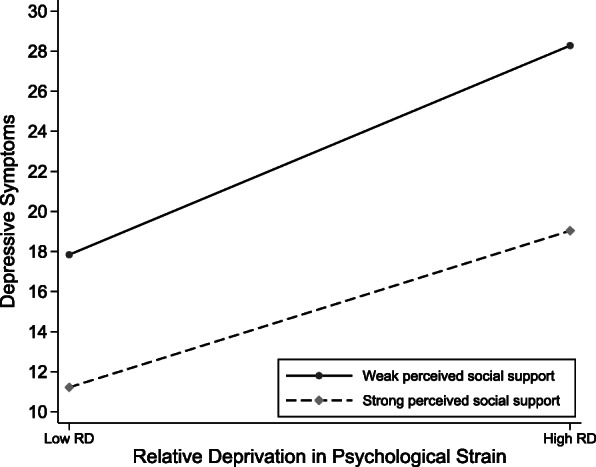
Graph of the moderating effects of perceived social support on relative deprivation in psychological strain

Second, although an increase in personal relative deprivation was generally associated with an increase in depressive symptoms, the positive effects of relative deprivation in income (Fig. 1) and relative deprivation in psychological strain (Fig. 2) were buffered by perceived social support (models 4 and 5). This indicates that not all individuals who experience personal relative deprivation are necessarily depressed but rather that there is a diversity of experience depending on the buffering effects of perceived social support. Cohen and Wills found that social support operates in such a way that perceived social support helps reduce stress assessment in stressful situations, alleviating the psychological trauma caused by stressful events [29]. Consistent with the buffering hypothesis, perceived social support serves as a moderator, absorbing part of the relative deprivation strain and protecting against depressive symptoms.

## Limitations and future research directions

This study has some limitations that can provide directions for future research. Its major limitation is its use of a cross-sectional design, which allows statistical moderation to be identified but does not provide definitive evidence on whether the assumed cause precedes the assumed effect or whether the relationship is caused by a third factor. Thus, findings of this study provide only preliminary evidence of the moderation mechanism, such that the relationship between depressive symptoms and personal relative deprivation could be moderated by perceived social support. Future studies could further substantiate the relationship of a comprehensive measure of subjective personal relative deprivation with depressive symptomology and examine exactly which sub-constructs of social support drive the buffering effect for the above relationship. This would be an important contribution to the literature on inequality and mental health. Additionally, the current sample is not representative of the general population, being younger (aged 17–59), more educated, and more urban. Our recruitment strategy thus limited the generalizability of our results. Recruiting participants online also has the potential sampling bias which might affect study results and conclusions. For example, the mean score of CES-D depression is 19.78 in our study, which is relatively higher than other populations in previous research [38, 39]. In addition, participants in our study may have lower levels of deprivation compared to the general population. The mean score of relative deprivation measured by Liu and colleagues with the Psychological Strain Scale (PSS) using an employee sample from urban China was between 23.28 and 24.45 [40]. Future work should investigate the effects of personal relative deprivation in a larger sample and examine the role of other variables (such as self-esteem) in the relationship between personal relative deprivation and depressive symptoms.

## Conclusions

The findings of this study demonstrate the importance of relative deprivation for psychological strain and income in explaining how socioeconomic indices correlate with depressive symptoms. The results of this study also indicate the need to acknowledge the interaction between perceived social support and personal relative deprivation in influencing depression. This research provides several implications in this regard: first, psychological counselors and social workers should pay closer attention to depressed individuals who experience more relative deprivation in perceived social support and use multiple methods, including group counseling, to help people who meet this profile buffer the stress from psychological strain to reduce their depressive symptoms; second, social and political action are imminently necessary to reduce inequality in China, as it is important to allocate wealth impartially to improve support well-being and mental health.

## Supplementary Information

**Additional file 1.** Questionnaire on Perception of Daily Life.

## Data Availability

The datasets used and/or analyzed during the current study are available from the corresponding author on reasonable request. The survey is available as supplementary material.

## Abbreviations

RD: Relative Deprivation
PSS: Psychological Strain Scale
CES-D: The Center for Epidemiologic Studies Depression Scale
MSPSS: The Multidimensional Scale of Perceived Social Support
ISEI: The International Socioeconomic Index
IRLS: The iteratively reweighted least square
OLS: The ordinary least square
