# Supplementary material for: Feeling matters: perceived social support moderates the relationship between personal relative deprivation and depressive symptoms
Source: BMC Psychiatry. 2021 Jul 12;21:345. doi: 10.1186/s12888-021-03334-8 (PMC8273951; doi:10.1186/s12888-021-03334-8)
Supplement: Supplementary file 1 — Additional file 1. Questionnaire on Perception of Daily Life. [file 12888_2021_3334_MOESM1_ESM.docx]

**Questionnaire on Perception of Daily Life**

Serial Number: Spring 2017 __________

The purpose of this survey is to better understand your viewpoints of some social concepts and social issues, as well as your individual feelings in some aspects of daily life. Please answer the questions according to your real thinking or feeling. Your participation is very important to our study, although voluntary. This questionnaire is also anonymous, and you are encouraged to answer every single question true to you. You have the right to refuse or stop the survey at any time. The information you have provided is absolutely confidential, and therefore, do not leave any personal information on the questionnaire.

**Part 1: Demographics**

1. Sex: ①= Male; ②= Female.

2. Birth： year month

3. Your highest level of education

①= None or dropout from primary school; ②= Primary school; ③= Middle

School; ④= Vocational High School ⑤= High school; ⑥= Technical Secondary School; ⑦= Technical School ⑧= Junior College ⑨= College; ⑩= Master Degree and Above

4. Marital status:

①. Single ②. Cohabitation ③. Married ④. Remarried ⑤. Separated But Not Divorced ⑥. Divorced ⑦. Widowed

5. Your religious belief:

①= None of religion; ②= Taoism; ③= Muslim; ④= Christian; ⑤= Catholicism; ⑥= Buddhism; ⑨= Others

6. Household registration (*Hukou*) status:

①. Rural *Hukou* ②. Urban *Hukou*

7. Last year，Your average monthly income is RMB。

8. How do you rank your family's economic situation compared with others in Beijing?

①=Very good; ②= Relatively good; ③= Average; ④= Relatively bad; ⑤= Very bad.

9. Occupation:

①.Farmer ②. Worker ③. Technicians or associate professionals（e.g. Engineer、Doctor） ④. Service or sales workers ⑤. Primary and secondary school teachers ⑥. College professors ⑦. Journalists, artists and sports workers ⑧. Managers ⑨. Legislator, senior officials ⑩. self-employed entrepreneurs . private entrepreneur . Soldier . unemployed . Other______

**Part 2: Individual Feelings**

**A Economy, income and social status**

Are you satisfied with your financial situation compare to the following groups?

|  | not at all satisfied | not very satisfied | generally satisfied | somewhat satisfied | completely satisfied |
| --- | --- | --- | --- | --- | --- |
| Friends | ① | ② | ③ | ④ | ⑤ |
| Colleagues | ① | ② | ③ | ④ | ⑤ |
| Occupation peers | ① | ② | ③ | ④ | ⑤ |
| Significant others | ① | ② | ③ | ④ | ⑤ |

**B MSPSS**

| Item | strongly disagree |  |  |  |  |  | strongly agree |
| --- | --- | --- | --- | --- | --- | --- | --- |
| There is a special person who is around when I am in need. | ① | ② | ③ | ④ | ⑤ | ⑥ | ⑦ |
| There is a special person with whom I can share my joys and sorrows | ① | ② | ③ | ④ | ⑤ | ⑥ | ⑦ |
| My family really tries to help me. | ① | ② | ③ | ④ | ⑤ | ⑥ | ⑦ |
| I get the emotional help and support I need from my family. | ① | ② | ③ | ④ | ⑤ | ⑥ | ⑦ |
| I get the emotional help and support I need from my family. | ① | ② | ③ | ④ | ⑤ | ⑥ | ⑦ |
| My friends really try to help me. | ① | ② | ③ | ④ | ⑤ | ⑥ | ⑦ |
| I can count on my friends when things go wrong. | ① | ② | ③ | ④ | ⑤ | ⑥ | ⑦ |
| I can talk about my problems with my family. | ① | ② | ③ | ④ | ⑤ | ⑥ | ⑦ |
| I have friends with whom I can share my joys and sorrows. | ① | ② | ③ | ④ | ⑤ | ⑥ | ⑦ |
| There is a special person in my life who cares about my feelings. | ① | ② | ③ | ④ | ⑤ | ⑥ | ⑦ |
| My family is willing to help me make decisions. | ① | ② | ③ | ④ | ⑤ | ⑥ | ⑦ |
| I can talk about my problems with my friends. | ① | ② | ③ | ④ | ⑤ | ⑥ | ⑦ |

**C Depression**

Below is a list of the ways you might have felt or behaved. Please tell me how often you have felt this way during the past week.

0 = Rarely or None of the Time (Less than 1 Day)

1 = Some or a Little of the Time (1-2 Days)

2 = Occasionally or a Moderate Amount of Time (3-4 Days)

3 = Most or All of the Time (5-7 Days)

| Item | 0 | 1 | 2 | 3 |
| --- | --- | --- | --- | --- |
| 1. I was bothered by things that usually don't bother me. |  | ① | ② | ③ |
| 2. I did not feel like eating; my appetite was poor. |  | ① | ② | ③ |
| 3. I felt that I could not shake off the blues even with help from my family or friends. |  | ① | ② | ③ |
| 4. I felt that I was just as good as other people. |  | ① | ② | ③ |
| 5. I had trouble keeping my mind on what I was doing. |  | ① | ② | ③ |
| 6. I felt depressed. |  | ① | ② | ③ |
| 7. I felt that everything I did was an effort |  | ① | ② | ③ |
| 8. I felt hopeful about the future. |  | ① | ② | ③ |
| 9. I thought my life had been a failure. |  | ① | ② | ③ |
| 10. I felt fearful. |  | ① | ② | ③ |
| 11. My sleep was restless |  | ① | ② | ③ |
| 12. I talked less than usual. |  | ① | ② | ③ |
| 13. I felt lonely. |  | ① | ② | ③ |
| 14. People were unfriendly. |  | ① | ② | ③ |
| 15. I enjoyed life. |  | ① | ② | ③ |
| 16. I had crying spells. |  | ① | ② | ③ |
| 17. I felt sad. |  | ① | ② | ③ |
| 18. I felt that people dislike me. |  | ① | ② | ③ |
| 19. I could not get “going”. |  | ① | ② | ③ |
| 20. I was happy. |  | ① | ② | ③ |

**Part 3：Strain and Suicide**

**A Kessler NCS and Attitude**

1. Have you ever seriously thought about killing yourself? ①=Yes =No

2．Have you ever made a plan for committing suicide? ①=Yes =No

3．Have you ever attempted suicide? ①=Yes =No

**B PSS**

Please read each of them carefully and respond truthfully by 1 (never, it’s not me at all), 2 (rarely, it’s not me), 3 (maybe, I’m not sure), 4 (often, it’s like me), and 5 (Yes, strongly agree and it’s exactly me). There are not right or wrong answers.

**Value**

|  |  |  |  |  |  |
| --- | --- | --- | --- | --- | --- |
| I am often confused about what life means to me. | ① | ② | ③ | ④ | ⑤ |
| I am unsure about what is right and wrong regarding some things in my daily life. | ① | ② | ③ | ④ | ⑤ |
| I don’t know why my thoughts are often different from others. | ① | ② | ③ | ④ | ⑤ |
| My parents and my best friends (peers) sometimes have different views on certain things, and I always find it difficult to deal with them. | ① | ② | ③ | ④ | ⑤ |
| I don’t know if women should have the same rights that men do. | ① | ② | ③ | ④ | ⑤ |
| Between traditional and modern values, I don’t know what I should follow. | ① | ② | ③ | ④ | ⑤ |
| Between chastity and sexual liberty, I don’t know what I should do. | ① | ② | ③ | ④ | ⑤ |
| I am always troubled by some conflicting ideas. | ① | ② | ③ | ④ | ⑤ |
| I am not living in the way I want, and I feel bad about it. | ① | ② | ③ | ④ | ⑤ |
| The traditional values are always opposite to what I have learned from school, I cannot make a choice what to believe. | ① | ② | ③ | ④ | ⑤ |

**Aspiration**

|  |  |  |  |  |  |
| --- | --- | --- | --- | --- | --- |
| Society is not fair to me. | ① | ② | ③ | ④ | ⑤ |
| I wish I were living in a better family, but I cannot realize it according to some reasons. | ① | ② | ③ | ④ | ⑤ |
| I wish I had a chance to get more education, but I cannot realize it according to some reasons. | ① | ② | ③ | ④ | ⑤ |
| I wish I had more power in my life, but I cannot realize it according to some reasons. | ① | ② | ③ | ④ | ⑤ |
| Many people have got in the way of my success | ① | ② | ③ | ④ | ⑤ |
| My life quality is not as good as it was before. | ① | ② | ③ | ④ | ⑤ |
| I wish I could change my current living condition, but I cannot. | ① | ② | ③ | ④ | ⑤ |
| I wish I could achieve the highest goal in my life, but I cannot. | ① | ② | ③ | ④ | ⑤ |
| wish I could be successful, but there are too many obstacles in my life. | ① | ② | ③ | ④ | ⑤ |
| I wish I had fewer burdens in my life, but I have to deal with so many responsibilities every day. | ① | ② | ③ | ④ | ⑤ |

**Relative deprivation**

|  |  |  |  |  |  |
| --- | --- | --- | --- | --- | --- |
| Compared to others in my neighborhood, I am a poor person. | ① | ② | ③ | ④ | ⑤ |
| Compared to other families in my community, my family is poor. | ① | ② | ③ | ④ | ⑤ |
| I believe I am good enough, but I am not satisfied with the treatment from others. | ① | ② | ③ | ④ | ⑤ |
| My family does not have the money to support me to go to school. | ① | ② | ③ | ④ | ⑤ |
| I cannot go to society as much as people around me can, because I am a poor person. | ① | ② | ③ | ④ | ⑤ |
| I have the same qualities as some of my colleagues, but they are paid much more than I am. | ① | ② | ③ | ④ | ⑤ |
| People around me are living in better apartments or houses. | ① | ② | ③ | ④ | ⑤ |
| I work hard and my performance is excellent, but I am not appreciated and promoted as are others who did not do their jobs so good. | ① | ② | ③ | ④ | ⑤ |
| Compared to others, it is more difficult for me to make money. | ① | ② | ③ | ④ | ⑤ |
| I have worked too much and gained too little. | ① | ② | ③ | ④ | ⑤ |

**Coping**

|  |  |  |  |  |  |
| --- | --- | --- | --- | --- | --- |
| Face is so important to me that I will do everything to protect my public image, even suicide. | ① | ② | ③ | ④ | ⑤ |
| I cannot handle too many things at the same time. | ① | ② | ③ | ④ | ⑤ |
| When confronted with some crisis, my head usually turns blank. | ① | ② | ③ | ④ | ⑤ |
| I always to do things as I like, without thinking of the consequence. | ① | ② | ③ | ④ | ⑤ |
| I cannot forget unpleasant experiences, and the more I think, the worse my feelings are. | ① | ② | ③ | ④ | ⑤ |
| Even with small problems, I sometimes feel low and cannot get going. | ① | ② | ③ | ④ | ⑤ |
| When I have problems, I feel difficult to fall asleep and lose my appetite. | ① | ② | ③ | ④ | ⑤ |
| When I have difficulties in what I am doing, I usually give up the task. | ① | ② | ③ | ④ | ⑤ |
| When I have a problem, I always stay alone and away from others. | ① | ② | ③ | ④ | ⑤ |
| In dealing with things, I often feel out of control and not able to catch up. | ① | ② | ③ | ④ | ⑤ |

**Thank you！**
